# Longitudinal Trends in Illness Perception and Depression during Adjuvant Breast Cancer Endocrine Therapy: A Prospective Observational Study

**DOI:** 10.3390/healthcare9091223

**Published:** 2021-09-16

**Authors:** Seul Ki Park, Yul Ha Min, Sae Byul Lee

**Affiliations:** 1 Red Cross College of Nursing, Chung-Ang University, Seoul 06974, Korea; gomtty3@cau.ac.kr; 2 College of Nursing, Kangwon National University, Chuncheon-si 24341, Korea; 3 Division of Breast Surgery, Department of Surgery, University of Ulsan College of Medicine, Asan Medical Center, Seoul 05505, Korea; newstar153@ulsan.ac.kr

**Keywords:** breast cancer, illness perception, depression, adjuvant endocrine therapy, women’s health

## Abstract

This study aimed to identify the changes in the illness perceptions and depression of women with breast cancer, undergoing AET, at three time points (i.e., before initiating AET, 3 months follow-up, and 12 months follow-up). We investigated the interaction effects of their demographic and clinical characteristics on illness perception changes over time. Furthermore, factors including the patient’s characteristics and illness perceptions associated with depressive symptoms 1 year after starting AET were explored. Illness perception and depressive symptoms were assessed with the brief illness perception questionnaire and the Center for Epidemiologic Studies Depression Scale, in a prospective study of 150 women. The changes in illness perceptions and depression between the three time points were analyzed using repeated measures ANOVA. The factors associated with depressive symptoms were identified using regression analysis. Illness perception improved overall over the 12 months. However, more patients perceived their illness as chronic, experienced more symptoms, and developed negative beliefs that treatment could not control their disease. Patients’ depressive symptoms decreased significantly. Depression at the baseline, cancer stage, and the perception of personal control were highly associated with depression after 12 months. These findings suggest that healthcare providers should offer appropriate interventions to patients, for managing symptoms, having a positive belief that treatment can control their disease, and preventing long-term depressive symptoms.

## 1. Introduction

Globally, breast cancer is the most prevalent type of cancer, and the leading cause of mortality among women, with 2,090,000 new cases and 627,000 deaths in 2018 [1]. Among adult women in Korea, breast cancer is the most common type of malignancy, with 23,500 incidences in 2018 [2]. Approximately 50–78% of breast cancer patients in Korea are hormone receptor-positive [2], and they often receive long-term (i.e., 5 or more years) adjuvant endocrine therapy (AET) upon completion of primary therapy (e.g., surgery, radiation, or chemotherapy), to further reduce their cancer recurrence risk and increase their overall survival [3,4].

Although AET can reduce the risk of recurrence and death from breast cancer in women, it is associated with several physical and psychological side effects, such as hot flushes, night sweating, joint pain, and depression [5,6]. The prevalence of depression among patients undergoing AET was found to range between 30% [5] and 50% [7]. Evidence shows that depression in patients with breast cancer increases poor adherence to cancer treatment [8,9], cancer recurrence [10,11], and cancer-specific mortality [10]. Additionally, it is known that depression is associated with several other factors, including not only the patient’s socio-demographic (e.g., age, socio-economic status) and clinical characteristics (e.g., cancer stage, treatments) [12,13,14], but also their perception of the illness [15,16].

Illness perception is a concept described in the self-regulatory model of illness by Leventhal and colleagues. The model explains how individual symptoms and emotions experienced during a health threat or diagnosis influence the perception of illness, and guide the subsequent coping behavior [17,18]. People’s illness perceptions are proposed as major determinants of their health outcomes. According to this model, after being diagnosed with breast cancer, the patients create cognitive representations of their illness, consisting of the following five dimensions: beliefs regarding illness identity (the symptoms the patient associates with the illness), cause (personal ideas about etiology), control (their belief in their ability to control the illness), consequences (their view about the impact of the illness on his/her life), and timeline beliefs (perceptions of the duration of the illness). The emotional dimensions of illness representations incorporate people’s level of concern about disease and its perceived emotional impact.

Research has demonstrated that the illness perceptions of women with breast cancer differ according to the patient’s demographic and clinical factors. Better-educated women recognized more symptoms and younger women had more confidence in their ability to control the illness [19]. Patients who were not working perceived more-severe consequences, less treatment control, and stronger emotional impacts [20]. Evidence also has shown that illness perception in breast cancer patients is suggested to be one of the significant causes of depression. For example, illness perception is strongly associated with depression in early stage breast cancer survivors, within 6 months to 12 years follow-up [15]. The study found that patients who had a lower positive illness perception exhibited more depression.

Illness perception or depression may change over time, after cancer diagnosis or during treatment. Although one previous study showed that illness perceptions and depression remained relatively stable at diagnosis and at 6 months post-diagnosis among breast cancer patients [21], other studies found that distress or depression changed over time, after surgery [22,23] or during different intermittent periods of chemotherapy [24]. Moreover, the variation in illness perception could have an effect on distress after diagnosis or during treatment. For example, Millar et al. [23] analyzed an individual variation in psychological morbidity in the year following surgery for breast cancer, and found that it was predicted by the patient’s immediate postoperative state of distress, her perception of the impact of the symptoms, and the timeline of the disease. Although the number of studies about illness perception or depression in breast cancer patients undergoing AET is increasing, most of the research has been limited to cross-sectional studies. It is important to evaluate the patterns of change in illness perception and depression during the period of AET, and the potential association between them, for implementing customized emotional interventions for each AET treatment period.

Given this background, the aim of this prospective observational study was to explore patients’ changes in illness perception and depression over a 12-month period, after having started AET, and to investigate the interaction effects of their demographic and clinical characteristics on illness perception changes. Furthermore, factors including the patient’s characteristics and illness perceptions associated with depressive symptoms 1 year after starting AET were explored.

## 2. Materials and Methods

### 2.1. Design

A prospective observational study design was used.

### 2.2. Sample and Setting

Patients were recruited at the Asan Medical Center, a university-affiliated hospital in Seoul, South Korea. A total of 556 consecutive women who had been histologically confirmed to have breast cancer at the Asan Medical Center were screened for eligibility from November 2016 to April 2017, at the time they were admitted for surgery. Inclusion criteria were as follows: cases with hormone receptor-positive breast cancer, an age of ≥20 years at diagnosis, and definitive surgery followed by AET, irrespective of chemotherapy. Women with distant metastases at diagnosis (stage IV breast cancer), local or regional recurrent tumors, or a medical history of psychiatric or neurologic illness were excluded. Prior to starting AET, patients were asked to provide the baseline data set (T1) by filling out a self-report survey, including the illness perception questionnaire and depression scale (see Section 2.3). After the baseline measurement, the patients completed the survey at 3 or 6 (T2) and 12 or 18 (T3) months after starting AET. Among 370 eligible patients, 210 patients consented to participate in this study (declined to participate: 137, unable to contact: 23). Among these remaining 210 patients, 60 patients were excluded (33 withdrawal of consent during the research, 8 lost to follow-up, 2 stopped AET, 5 with recurrent breast cancer, and 12 did not respond at any one of the three time points). Finally, 150 patients completed all questionnaires at all three points, and their data were included in this analysis.

### 2.3. Data Collection

The demographic and clinical characteristics of the participants were extracted from electronic medical records with their consent at T1. The demographic characteristics were age, educational level, marital status, employment status, family history of cancer, and family history of breast cancer. The clinical characteristics were menopausal status, cancer stage, type of surgery, history of chemotherapy and radiation therapy, and type of hormone therapy agent.

Illness perception was assessed with the Korean version of the brief illness perception questionnaire (BIPQ) [25] at T1, T2, and T3. The BIPQ consists of eight questions that measure eight dimensions of illness perceptions for assessing cognitive and emotional representations of the illness. The questions were related to the following: (i) consequences (how much does your illness affect your life?); (ii) timeline (how long do you think your illness will continue?); (iii) personal control (how much control do you feel you have over your illness?); (iv) treatment control (how much do you think your treatment can help your illness?); (v) identity (how much do you experience symptoms from your illness?); (vi) concern (how concerned are you about your illness?); (vii) coherence (how well do you feel you understand your illness?); and (viii) emotions (how much does your illness affect you emotionally?). Each question is rated from 0 to 10. Higher scores indicate more negative illness representations, except for questions iii, iv, and vii, where higher scores indicate a more positive illness perception [26].

Depression was assessed by the Center for Epidemiologic Studies Depression Scale (CES-D) [27] at T1, T2, and T3. This is a 20-item self-report scale measuring the presence and degree of depressive symptoms. It includes the following six components: depressed mood; feelings of guilt and worthlessness; feelings of helplessness and hopelessness; psychomotor retardation; loss of appetite; and sleep disturbance. Respondents indicate how often within the last week they experienced the symptoms, responding with the following: “rarely or none of the time” (0); “some or little of the time” (1); “occasionally or a moderate amount of time” (2); and “most or all of the time” (3). The scores for the 20 items are added, resulting in a range of possible total scores from 0 to 60. We used total scores of this scale for analyzing depression of patients.

### 2.4. Data Analysis

The demographic and clinical characteristics of the patients were analyzed descriptively. To explore changes in illness perception and depression over time, repeated measures ANOVA was used. To identify interaction effects between demographic and clinical characteristics and changes in illness perception over time, repeated measures ANOVA was also used.

To identify factors associated with depression at T3, multiple regression analysis was used. We conducted regression analyses using cross-sectional and prospective analyses. A cross-sectional analysis was undertaken to identify whether the illness perceptions measured at T3 were associated with depression at T3. In addition, two prospective analyses were conducted to identify whether the illness perceptions measured before starting AET (T1) and 3 months later (T2) were associated with depression at T3. The prospective analysis is meaningful in light of the result of previous research [23] showing long-term outcome to be significantly predicted by illness perception before 12 months. We used demographic and clinical characteristics and depression at baseline, as well as illness perceptions as independent variables to adjust for their effect in both the cross-sectional and the prospective analyses.

## 3. Results

### 3.1. Characteristics of the Patients

The demographic and clinical characteristics of the patients are shown in Table 1. The mean age was 47.20 (SD = 8.84) years, and most were married (86.7%). Overall, 58.0% had at least a bachelor’s degree and 51.3% were currently employed. A total of 35.3% and 8.7% reported having a family history of any cancer and breast cancer, respectively. About a third of the patients (30.0%) were menopausal. Most had stage I, II, or III (83.3%) cancer. Regarding surgery, 84.7% had received conservation surgery. Regarding previous treatments for breast cancer, 30.7% and 87.3% had received chemotherapy and radiation therapy, respectively. A total of 77.3% and 22.7% of the patients were taking tamoxifen and aromatase inhibitor as hormone therapy agents, respectively.

### 3.2. Changes in Illness Perceptions and Depression between the Three Time Points

As shown in Table 2, the repeated measures ANOVA revealed statistically significant changes in all the dimensions of illness perception and depression between the three time points, except for the perception of personal control dimension. More specifically, there were significant increases in the perception of timeline (*p* < 0.001), identity (*p* = 0.038), and coherence (*p* = 0.025). There were also significant decreases in the perception of consequences (*p* < 0.001), treatment control (*p* = 0.002), concern (*p* = 0.031), and emotions (*p* = 0.021). The depression score declined significantly over the follow-up period (*p* = 0.003).

### 3.3. Effect of Demographic and Clinical Characteristics on Illness Perception Changes

Repeated measures ANOVA within subjects was conducted to explore the interaction effects of demographical and clinical characteristics on the illness perception changes. Table 3 shows the significant interaction effects between demographic and clinical characteristics, and time on the change in illness perception dimensions.

More specifically, being of an age under 40 had a significant interaction effect on increased perceptions of timeline (*p* = 0.027). Being employed had a significant interaction effect on decreased treatment control (*p* = 0.021). Being premenopausal (*p* = 0.037) or not having had chemotherapy (*p* = 0.037) had a significant interaction effect on increased perceptions of symptoms.

### 3.4. Factors Associated with Depression after 12 Months

Regression analyses were conducted to determine the factors that predicted depression at 12 months follow-up. The outcomes of a cross-sectional and two prospective analyses are shown in Table 4. In the cross-sectional analysis, depression at 12 months was predicted by the depression score at the baseline, as well as education level, cancer stage, and chemotherapy, and also the patients’ perceptions of personal control, treatment control, identity, and emotions, measured at 12 months. In the prospective analysis, depression at 12 months was predicted by the depression score at the baseline, cancer stage, and the patients’ perceptions of consequences and personal control at the baseline. However, any illness perceptions measured at 3 months were not significant factors associated with depression at 12 months. Depression at 12 months was only predicted by the depression score at the baseline and the cancer stage.

## 4. Discussion

This study examined the changes in illness perception and depression among patients with breast cancer, over 12 months after starting AET, and explored factors associated with depressive symptoms.

The patients’ illness perceptions changed significantly over time. After 12 months, they experienced lower perceived consequences of the illness on their life and were more likely to perceive their illness as chronic. In addition, changes indicated that patients were less concerned about their illness, had a better understanding of it, and it affected them less emotionally, which represent significant positive changes. However, patients experienced more symptoms and developed negative beliefs that treatment could not control their disease. These results are different from those of previous longitudinal studies, which found that there was little meaningful change in illness perceptions over time, from diagnosis to 6 months post-diagnosis [21] and 1 year after surgery, among breast cancer patients [23]. This inconsistency could be because previous studies focused on patients undergoing surgery, which shows dramatic change in a short period of time, or receiving intermittent chemotherapy, whereas this study focused on patients who had to take a drug daily for 5 years, which is a much longer period. Based on the finding of a previous study that symptoms in women undergoing AET persisted throughout 5 years of treatment [28], patients are likely to experience persistent side effects as the duration of the treatment increases. Side effects are the major barrier to non-adherence to AET, in both patient-reported and physician-reported surveys [29]. Therefore, it can be difficult for patients to be sure whether they should take this drug with these side effects for a long time. Moreover, AET is a prophylactic to prevent the recurrence of breast cancer, and is not a treatment that can control symptoms and immediately confirm its effects, such as antihypertensive drugs [30]. This might cause patients to develop the negative belief that the treatment could not cure the disease.

We also found that being premenopausal or not having had chemotherapy had a significant interaction effect on increased perceptions of symptoms. Tamoxifen is mainly prescribed to premenopausal women, whereas aromatase inhibitors are only prescribed to postmenopausal women. Tamoxifen is associated with a range of side effects, including hot flushes, night sweats, weight gain, insomnia, joint pain, and vaginal dryness. Compared to other breast cancer cases only, those taking tamoxifen were 2.6 times more likely to report menopausal symptoms [31]. Even after chemotherapy, women with breast cancer frequently experience severe menopausal symptoms [32]. As a result, whereas the patients who were premenopausal or had not received chemotherapy in this study may have experienced menopausal symptoms that they had never experienced before AET, the patients who were postmenopausal or had received chemotherapy may have easily adapted to the menopausal symptoms, as they are likely to have experienced them before AET. However, since the presence of menopause-related symptoms, both prior to AET initiation [33] and during AET [34], can affect non-adherence to AET, appropriate interventions to manage the associated symptoms will be required.

Being employed had a significant interaction effect on the perceptions of decreased treatment control. This finding has important implications for supporting women who are employed. Previous studies showed that women who are employed are twice as likely to experience menopausal symptoms related to AET [28], and that the symptoms cause difficulty at work, and may negatively impact work performance [35]. Employed patients may have low beliefs about their medications, due to difficulty in maintaining strict adherence to AET and in managing its side effects. Therefore, more targeted support for employed patients is needed, regarding symptom management and education on the necessity of AET.

Patients’ depression scores decreased significantly over time. This result is consistent with previous studies that examined the change in depression over 12 months after surgical treatment [22], and during intermittent periods of adjuvant chemotherapy [24]. The reason for such change might be that, as the treatment period increases, patients gradually accept reality and gain a deeper understanding of their disease and its associated treatment [24].

We explored the factors associated with depression at 12 months follow-up. Initial depression, cancer stage, and personal control were significant predictors of an increase in depression, both in the cross-sectional and prospective analyses. The influence of depressive symptoms before diagnosis or treatment on depressive symptoms after diagnosis or treatment has been explored in other studies. These reported that depressive symptoms before the diagnosis of breast cancer have an effect on experiencing depressive symptoms 12 months after surgical treatment [22], and that patients with previous episodes of depression had a higher risk of depression if they had been diagnosed with breast cancer [36]. Since depressive symptoms increase poor adherence to cancer treatment [8,9] and cancer recurrence [10,11], this highlights the need to assess the patient’s depressive symptoms when starting AET, and to offer psychological interventions across the duration of treatment where necessary, to prevent long-term depressive symptoms.

An unexpected finding was that high levels of personal control measured before initiating AET was associated with higher levels of depression at the 12 months follow-up, whereas lower perceptions of control over the illness at 12 months was also associated with a higher level of depression at 12 months. The reason for such difference is unclear. Although many previous studies support the idea that having a strong sense of personal control relates to better emotional well-being, such as less depression [37,38], some have been convinced that perceived personal control is more likely to have negative, rather than positive, effects. For example, Rodin argued that as patients’ physical problems become more severe and chronic, greater perceived control over these problems can yield more stress, worry, and self-blame [39]. As personal control often places heavy demands on patients, in the form of high investments of time, effort, resources, and the risk of the consequences of failure, patients with a perception of greater personal control may suffer from self-blame for having caused their health-related problems, leading to poor care [39]. Therefore, patients who had a higher sense of personal control at the beginning of AET, and experienced side effects during the course of their treatment, may feel guilty for not being able to control these symptoms, and the situation could have led to the increased depression found in this study. Another possible reason is that some patients might have had an unrealistically high sense of control over the illness at the baseline, maybe because they did not know much about it yet. A year later, their sense of control has fallen to a more realistic level, and it is possible that this harsh realization might lead to more depression.

The findings of this study must be evaluated in the context of some limitations. This study recruited a nonrandomized sample of participants with breast cancer from a single center, which may limit the generalizability of the findings. In addition, the follow-up period for assessing illness perception and depression was limited to 1 year after starting AET. A longer follow-up time may have revealed further or different changes in illness perception and depression. Meanwhile, a recent study revealed that the subjective feeling of depression can be measured by an objective method, such as a heart rate variability measurement [40]. Future studies may assess depressive symptoms using objective biological substrates, as well as subjective self-report investigations. Therefore, additional long-term multi-site follow-up studies assessing patients with breast cancer should be performed, to investigate the changes and associations between illness perception and depression.

## 5. Conclusions

This prospective study was conducted to identify the changes in the illness perceptions and depression of women with breast cancer, undergoing AET, at three time points (i.e., before initiating AET, 3 months follow-up, and 12-month follow-up). An overall improvement of illness perception has been shown over the 12 months, indicating positive changes. However, over the 12 months, patients were more likely to perceive their illness as chronic, to have experienced more symptoms, and to have developed negative beliefs that treatment could not control their disease. Being employed had a significant interaction effect on decreased perceived treatment control, and being premenopausal or not having had chemotherapy had a significant interaction effect on increased perceptions of symptoms. Patients’ depression scores decreased significantly over time. Depression at the baseline, cancer stage, and perception of personal control are highly associated with depression at 12-months follow-up.

The results of this study suggest that healthcare providers should frequently educate patients on the necessity of AET, so that they can have a positive belief that treatment can control their disease. Furthermore, appropriate interventions for symptom management should be provided. Additionally, it is important to screen women with depressive symptoms before initiating AET, and offer them psychological interventions to prevent long-term depressive symptoms.

## Figures and Tables

**Table 1:** Demographic and clinical characteristics of the patients (n = 150).

Characteristic		Mean (SD ^1^) or n (%)
Age (years)		47.20 (8.84)
	≤40	27 (18.0)
	>40	123 (82.0)
Education level	High school or below	63 (42.0)
	College and above	87 (58.0)
Marital status	No	20 (13.3)
	Yes	130 (86.7)
Employment status	No	73 (48.7)
	Yes	77 (51.3)
Family history of cancer	No	97 (64.7)
	Yes	53 (35.3)
Family history of breast cancer	No	137 (91.3)
	Yes	13 (8.7)
Menopausal status	No	105 (70.0)
	Yes	45 (30.0)
Cancer stage	In situ	25 (16.7)
	Invasive (Stage I, II, or III)	125 (83.3)
Type of surgery	Mastectomy	23 (15.3)
	Conservation	127 (84.7)
Chemotherapy	No	104 (69.3)
	Yes	46 (30.7)
Radiation therapy	No	19 (12.7)
	Yes	131 (87.3)
Hormone therapy agent	Tamoxifen	116 (77.3)
	Aromatase inhibitor	34 (22.7)

^1^ Standard deviation.

**Table 2:** Illness perception and depression at three points (T1, T2, and T3).

	T1 ^1^	T2 ^1^	T3 ^1^	F Value	*p* Value
*Illness perception*					
Consequences	5.90 (2.54)	5.26 (2.46)	4.83 (2.67)	13.911	<0.001
Timeline	4.62 (2.88)	4.59 (2.64)	5.89 (2.19)	14.169	<0.001
Personal control	5.73 (2.20)	5.77 (2.06)	5.89 (2.19)	0.337	0.700
Treatment control	8.25 (1.89)	7.78 (1.98)	7.65 (1.84)	6.307	0.002
Identity	3.63 (2.72)	3.87 (2.57)	4.24 (2.47)	3.418	0.038
Concern	5.75 (2.76)	5.65 (2.63)	5.28 (2.66)	3.525	0.031
Coherence	5.62 (2.38)	6.03 (2.13)	6.09 (2.33)	3.739	0.025
Emotions	5.26 (2.79)	4.79 (2.54)	4.73 (2.49)	3.897	0.021
*Depression*	16.72 (9.97)	14.68 (9.13)	14.41 (9.02)	5.836	0.003

^1^ Data presented as mean (SD).

**Table 3:** The interaction effects of demographic and clinical characteristics on illness perception changes.

Illness Perception	Factor		T1 ^1^	T2 ^1^	T3 ^1^	F Value	*p* Value
Timeline	Age	≤40	4.56 (3.23)	4.96 (2.62)	5.59 (3.02)	3.665	0.027
		>40	4.63 (2.81)	4.50 (2.64)	4.24 (2.62)		
Personal control	Education level	High school or below	6.10 (2.13)	5.44 (2.17)	5.79 (1.95)	4.270	0.017
		College and above	5.46 (2.23)	6.01 (1.96)	5.97 (2.36)		
Treatment control	Employment status	No	8.10 (2.03)	8.10 (1.88)	7.89 (1.69)	3.896	0.021
		Yes	8.39 (1.75)	7.48 (2.04)	7.43 (1.96)		
Identity	Menopausal status	No	3.51 (2.69)	4.16 (2.50)	4.30 (2.39)	3.436	0.037
		Yes	3.89 (2.81)	3.20 (2.62)	4.09 (2.64)		
	Chemotherapy	No	3.07 (2.58)	3.72 (2.47)	3.91 (2.42)	3.442	0.037
		Yes	4.89 (2.63)	4.22 (2.76)	4.98 (2.43)		
Concern	Radiation therapy	No	6.00 (2.91)	5.21 (2.59)	6.05 (3.05)	3.095	0.047
		Yes	5.72 (2.75)	5.72 (2.64)	5.17 (2.59)		
Emotions	Chemotherapy	No	5.13 (2.81)	5.07 (2.39)	4.62 (2.46)	5.653	0.004
		Yes	5.54 (2.75)	4.15 (2.77)	4.98 (2.54)		

^1^ Data presented as mean (SD).

**Table 4:** Cross-sectional and prospective regression analyses to predict depression at 12 months follow-up.

	Adjusted R^2^	ß	t	*p* Value
***Cross-sectional analysis***				
**IP at 12 months follow-up**	0.544			
Depression_baseline		0.411	6.170	<0.001
Education level		−0.137	−2.291	0.024
Cancer stage		0.168	2.672	0.009
Chemotherapy		0.140	−2.240	0.027
IP ^1^ Personal control_12 months		−0.188	−2.797	0.006
IP Treatment control_12 months		−0.151	−2.340	0.021
IP Identity_12 months		0.185	2.682	0.008
IP Emotions_12 months		0.208	2.973	0.004
***Prospective analysis***				
**IP at baseline**	0.408			
Depression_baseline		0.573	7.868	<0.001
Cancer stage		0.173	2.587	0.011
IP Consequences_baseline		0.187	2.625	0.010
IP Personal control_baseline		0.152	2.237	0.027
**IP at 3 months follow-up**	0.366			
Depression_baseline		0.609	8.785	<0.001
Cancer stage		0.174	2.513	0.013

^1^ Illness perception.

## Data Availability

The datasets used and/or analyzed during the current study are available from the corresponding author on reasonable request.

## References

[1] Bray F., Ferlay J., Soerjomataram I., Siegel R.L., Torre L.A., Jemal A. (2018). Global cancer statistics 2018: GLOBOCAN estimates of incidence and mortality worldwide for 36 cancers in 185 countries. CA Cancer J. Clin..

[2] Society KBC (2020). Breast Cancer Facts & Figures 2020.

[3] Davies C., Pan H., Godwin J., Gray R., Arriagada R., Raina V., Abraham M., Medeiros Alencar V.H., Badran A., Bonfill X. (2013). Long-term effects of continuing adjuvant tamoxifen to 10 years versus stopping at 5 years after diagnosis of oestrogen receptor-positive breast cancer: ATLAS, a randomised trial. Lancet.

[4] Burstein H.J., Temin S., Anderson H., Buchholz T.A., Davidson N.E., Gelmon K.E., Giordano S.H., Hudis C.A., Rowden D., Solky A.J. (2014). Adjuvant endocrine therapy for women with hormone receptor-positive breast cancer: American Society of Clinical Oncology Clinical Practice Guideline focused update. J. Clin. Oncol..

[5] Rosenberg S.M., Stanton A.L., Petrie K.J., Partridge A.H. (2015). Symptoms and symptom attribution among women on endocrine therapy for breast cancer. Oncologist.

[6] van Londen G.J., Beckjord E.B., Dew M.A., Cooper K.L., Davidson N.E., Bovbjerg D.H., Donovan H.S., Thurston R.C., Morse J.Q., Nutt S. (2014). Associations between adjuvant endocrine therapy and onset of physical and emotional concerns among breast cancer survivors. Support. Care Cancer.

[7] Haidinger R., Bauerfeind I. (2019). Long-Term side effects of adjuvant therapy in primary breast cancer patients: Results of a web-based survey. Breast Care.

[8] Bender C.M., Gentry A.L., Brufsky A.M., Casillo F.E., Cohen S.M., Dailey M.M., Donovan H.S., Dunbar-Jacob J., Jankowitz R.C., Rosenzweig M.Q. (2014). Influence of patient and treatment factors on adherence to adjuvant endocrine therapy in breast cancer. Oncol. Nurs. Forum.

[9] Mausbach B.T., Schwab R.B., Irwin S.A. (2015). Depression as a predictor of adherence to adjuvant endocrine therapy (AET) in women with breast cancer: A systematic review and meta-analysis. Breast Cancer Res. Treat..

[10] Wang X., Wang N., Zhong L., Wang S., Zheng Y., Yang B., Zhang J., Lin Y., Wang Z. (2020). Prognostic value of depression and anxiety on breast cancer recurrence and mortality: A systematic review and meta-analysis of 282,203 patients. Mol. Psychiatry.

[11] Chen S.J., Chang C.H., Chen K.C., Liu C.Y. (2016). Association between depressive disorders and risk of breast cancer recurrence after curative surgery. Medicine.

[12] Tsaras K., Papathanasiou I.V., Mitsi D., Veneti A., Kelesi M., Zyga S., Fradelos E.C. (2018). Assessment of depression and anxiety in breast cancer patients: Prevalence and associated factors. Asian Pac. J. Cancer Prev..

[13] Boing L., Pereira G.S., Araújo C., Sperandio F.F., Loch M., Bergmann A., Borgatto A.F., Guimarães A.C.A. (2019). Factors associated with depression symptoms in women after breast cancer. Rev. Saude Publica.

[14] Casavilca-Zambrano S., Custodio N., Liendo-Picoaga R., Cancino-Maldonado K., Esenarro L., Montesinos R., Bertani S., Fejerman L., Guerchet M., Vidaurre T. (2020). Depression in women with a diagnosis of breast cancer. Prevalence of symptoms of depression in Peruvian women with early breast cancer and related sociodemographic factors. Semin. Oncol..

[15] Kus T., Aktas G., Ekici H., Elboga G., Djamgoz S. (2017). Illness perception is a strong parameter on anxiety and depression scores in early-stage breast cancer survivors: A single-center cross-sectional study of Turkish patients. Support. Care Cancer.

[16] Gibbons A., Groarke A., Sweeney K. (2016). Predicting general and cancer-related distress in women with newly diagnosed breast cancer. BMC Cancer.

[17] Leventhal H., Benyamini Y., Brownlee S., Diefenbach M., Leventhal E.A., Patrick-Miller L., Robitaille C. (1997). Illness representations: Theoretical foundations. Percept. Health Illn..

[18] Leventhal H., Diefenbach M., Leventhal E.A. (1992). Illness cognition: Using common sense to understand treatment adherence and affect cognition interactions. Cogn. Ther. Res..

[19] Ma C., Yan J., Wu Y., Huang W. (2018). Illness perceptions of Chinese women with breast cancer and relationships with socio-demographic and clinical characteristics. Int. J. Nurs. Pract..

[20] Karabulutlu E.Y., Avcı İ.A., Karayurt Ö., Gürsoy A., Köşgeroğlu N., Tuna A., Ersin F., Arıkan F., Karaman S. (2019). Evaluation of illness perception of women with breast cancer in Turkey. Eur. J. Breast Health.

[21] McCorry N.K., Dempster M., Quinn J., Hogg A., Newell J., Moore M., Kelly S., Kirk S.J. (2013). Illness perception clusters at diagnosis predict psychological distress among women with breast cancer at 6 months post diagnosis. Psychooncology.

[22] Den Oudsten B.L., van Heck G.L., van der Steeg A.F., Roukema J.A., de Vries J. (2009). Predictors of depressive symptoms 12 months after surgical treatment of early-stage breast cancer. Psychooncology.

[23] Millar K., Purushotham A.D., McLatchie E., George W.D., Murray G.D. (2005). A 1-year prospective study of individual variation in distress, and illness perceptions, after treatment for breast cancer. J. Psychosom. Res..

[24] Zhang J., Zhou Y., Feng Z., Xu Y., Zeng G. (2018). Longitudinal trends in anxiety, depression, and quality of life during different intermittent periods of adjuvant breast cancer chemotherapy. Cancer Nurs..

[25] Shim E.J., Jeong D., Song Y.W., Lee S.H., Kim N.J., Hahm B.J. (2020). A network analysis of the Brief Illness Perception Questionnaire in patients with rheumatic diseases and human immunodeficiency virus infection. Psychol. Health.

[26] Broadbent E., Petrie K.J., Main J., Weinman J. (2006). The brief illness perception questionnaire. J. Psychosom. Res..

[27] Radloff L.S. (1977). The CES-D scale: A self-report depression scale for research in the general population. Appl. Psychol. Meas..

[28] Moon Z., Hunter M.S., Moss-Morris R., Hughes L.D. (2017). Factors related to the experience of menopausal symptoms in women prescribed tamoxifen. J. Psychosom. Obstet. Gynaecol..

[29] Paranjpe R., John G., Trivedi M., Abughosh S. (2019). Identifying adherence barriers to oral endocrine therapy among breast cancer survivors. Breast Cancer Res. Treat..

[30] Kim Y., Min Y.H. (2017). Validity and reliability of the Korean version of the Beliefs about Medicines Questionnaire-Specific for breast cancer patients on hormone therapy. J. Health Inf. Stat..

[31] Harris P.F., Remington P.L., Trentham-Dietz A., Allen C.I., Newcomb P.A. (2002). Prevalence and treatment of menopausal symptoms among breast cancer survivors. J. Pain Symptom. Manag..

[32] Dorjgochoo T., Gu K., Kallianpur A., Zheng Y., Zheng W., Chen Z., Lu W., Shu X.O. (2009). Menopausal symptoms among breast cancer patients 6 months after diagnosis: A report from the Shanghai Breast Cancer Survival Study. Menopause.

[33] Yusufov M., Nathan M., Wiley A., Russell J., Partridge A., Joffe H. (2021). Predictors of increased risk for early treatment non-adherence to oral anti-estrogen therapies in early-stage breast cancer patients. Breast Cancer Res. Treat..

[34] Simon R., Latreille J., Matte C., Desjardins P., Bergeron E. (2014). Adherence to adjuvant endocrine therapy in estrogen receptor-positive breast cancer patients with regular follow-up. Can. J. Surg..

[35] Griffiths A., MacLennan S.J., Hassard J. (2013). Menopause and work: An electronic survey of employees’ attitudes in the UK. Maturitas.

[36] Jacob L., Bleicher L., Kostev K., Kalder M. (2016). Prevalence of depression, anxiety and their risk factors in German women with breast cancer in general and gynecological practices. J. Cancer Res. Clin. Oncol..

[37] Thompson S.C., Sobolew-Shubin A., Galbraith M.E., Schwankovsky L., Cruzen D. (1993). Maintaining perceptions of control: Finding perceived control in low-control circumstances. J. Pers. Soc. Psychol..

[38] Burgess C., Morris T., Pettingale K.W. (1988). Psychological response to cancer diagnosis—II. Evidence for coping styles (coping styles and cancer diagnosis). J. Psychosom. Res..

[39] Rodin J. (1986). Aging and Health: Effects of the Sense of Control. Science.

[40] Jo H.-G., Shin N. (2021). Heart Rate Variability as an Early Objective Indicator of Subjective Feeling of Depression in Daily Life. Healthc. Inform. Res..

